# “You Know It, You Can Do It—Good Luck!”: Managing Music Performance Anxiety in the Context of Transforming Music Performance Ecosystems

**DOI:** 10.3390/bs15121696

**Published:** 2025-12-08

**Authors:** Natalija Šimunovič, Katarina Habe

**Affiliations:** Academy of Music, University of Ljubljana, 1000 Ljubljana, Slovenia; katarina.habe@ag.uni-lj.si

**Keywords:** music performance anxiety (MPA), ecosystemic approach, musical self-concept, music performance ecosystem, performer attunement

## Abstract

Music performance anxiety (MPA) can be examined within the framework of music performance ecosystems, wherein performers’ musical self-concept is shaped through complex social and cultural interactions. This research aims to identify ecosystemic interactions contributing to the emergence of MPA, and to promote a shift in its understanding, from a focus on individual symptomatology to the recognition of collective influences. A qualitative analysis was conducted using biographical-narrative interviews with 11 established musical performers (six female, five male), aged 23 to 62, representing a range of instruments, including violin, viola, cello, flute, piano, harp, and voice. Their experience encompasses solo, chamber, and orchestral performance, as well as pedagogical work, all rooted in the traditional educational framework of Western classical music. The analysis, based on the grounded theory methodology, highlights four key dimensions: the demanding stage; the development of psychological resilience in performers; the musical self-concept as a reflection of the performer’s experience; and performance as a process of transformation. The findings suggest that an ecosystemic perspective may support preventive interventions for MPA, particularly by reframing the traditional virtuoso performance model. Often internalized early in music education, this prestigious ideal continues to shape professional careers, placing heavy self-reflective demands on performers. To support healthier artistic development, music performance ecosystems can bridge the gap between skill acquisition and performer attunement. This is not merely a divide between learning and performing, but a structural loop in which training-oriented processes—such as analysis, self-criticism, and control—may hinder stage performance if not transformed into holistic, embodied execution.

## 1. Introduction

Music performance anxiety (MPA) is a pervasive challenge for musicians across educational stages and professional careers. Following Kenny (2009), we define MPA as marked and persistent anxious apprehension specifically tied to musical performance—arising in anticipation of, during, or immediately after performing, and expressed through affective, cognitive, somatic, and behavioral symptoms. MPA encompasses a spectrum of anxiety responses, which can manifest in different forms: (a) reactive anxiety, triggered by actual or perceived lack of resources for coping with performance demands, which may or may not impair performance quality; (b) maladaptive anxiety, in which heightened arousal diminishes performance capacity; and (c) pathological anxiety, which extends beyond performance contexts and aligns with generalized anxiety disorders. MPA occurs along a continuum of intensity, and when severe and persistent, it may be considered a disorder. Although classified in the DSM-5 as a subtype of social anxiety disorder (American Psychiatric Association, 2013), emerging evidence indicates that MPA merits recognition as a distinct condition (Kenny, 2023; Wiedemann et al., 2021).

A key limitation of dominant accounts is the reification of MPA as a static, individual disorder, thereby overlooking how sociocultural contexts and long-term musical enculturation shape its development, expression, and management (Herman & Clark, 2023; Pecen et al., 2018). Within such processes, the fostering or reinforcement of MPA cannot be attributed solely to an individual’s intrapsychic relation to musical activity; rather, the emergence and maintenance of MPA are continuously co-constituted and reshaped across educational, performance, and professional settings. For example, a conservatory oboist facing a final exam—after solitary drill and virtuosity-oriented learning—encounters a stuffy hall, a panel of fatigued jurors, and an unfamiliar accompanist; so, the oboist’s anxiety surges as the challenge outweighs their perceived competence (cf. O’Neill, 2017).

Therefore, locating the “problem” exclusively in the individual further isolates and internally divides the performer (Conable & Conable, 1995), while downplaying their embeddedness in a web of interactions—the very point emphasized by an ecosystem perspective (Haeckel, 1866; Tansley, 1935). A range of authors has already highlighted musical identity (Lamont, 1999, 2002), music learning (O’Neill, 2017), musical performing (Waters, 2007; Davis, 2011), as well as music cultures (Schippers & Grant, 2016) within the ecological paradigm. Some of these approaches (Lamont, 1999, 2002; O’Neill, 2017) assign a central role to the musical self-concept—grounded in self-reflection and social relatedness.

### 1.1. Musical Self-Concept Within Ecosystemic Perspectives on MPA

Spychiger (2017) conceptualizes musical self-concept as a multidimensional construct comprising academic (“What can I do?”) dimensions, that is, skills and knowledge in musical learning and performance, and non-academic (“Who am I?”) dimensions, encompassing bodily, emotional, cognitive, spiritual, and social facets of the individual’s musical experience and identity (see also Spychiger et al., 2009). The psychological construct of musical self-concept consistently appears in the literature as a construct related to MPA. A more positive and coherent musical self-concept is generally associated with lower levels of MPA, whereas fragmented or vulnerable self-concepts increase susceptibility to performance difficulties (Castiglione et al., 2018; Papageorgi et al., 2007). From an ecological perspective, this implies that interactional contexts matter: when environments emphasize specific social skills and display norms at the expense of holistic development, they may exacerbate anxiety rather than support resilience (Gaunt et al., 2021). In practice, such emphases include privileging the “notation argument” (Kivijärvi & Väkevä, 2020); marginalizing improvisation (Green, 2002; Sawyer, 2006; Solis & Nettl, 2009); insufficient cultivation of listening/imagining skills (Partti & Karlsen, 2010); instrumental technicism and narrow professionalism (Green, 2002; Westerlund et al., 2022); strongly competition-oriented programs (Papageorgi et al., 2007); weakened performer–audience connection (Juslin & Sloboda, 2010); avoidance or overemphasis of MPA in pedagogical discourse (Kenny, 2011); and the exaltation of a virtuoso mission at the expense of broader musicianship (MacDonald & Saarikallio, 2024). Such emphases function as contextual carriers of anxiety risk, rather than as supports for the holistic development of a musical self-concept conducive to health and well-being (MacDonald & Saarikallio, 2024; Spychiger, 2017; O’Neill, 2017).

Conversely, there is evidence that anxiety levels can be preventively reduced in school settings—for example, through the FRIENDS for Life program, which in a Swedish school-based pilot with primary school children was associated with decreases in depressive symptoms and behavioral difficulties, as well as reduced anxiety symptoms (see Ahlén et al., 2012). The program combined classroom discussions about anxiety, role-playing, written reflections, and peer-support activities with techniques such as recognizing and reshaping negative automatic thoughts, practicing relaxation and emotion-regulation skills, strengthening self-confidence, and developing assertive communication and conflict-resolution strategies. In this way, FRIENDS illustrates how preventive interventions can be effective through everyday classroom relationships and interactions, aligning with ecological approaches to prevention (Donovan & Spence, 2000; Kelly, 1967/1968, 2005; Webster, 2003).

Because we understand musical performance both as a situated onstage event (e.g., Banes & Lepecki, 2012; Clarke, 2005) and as a developmental social challenge (e.g., Borthwick & Davidson, 2002; Creech & Hallam, 2011; Davidson et al., 1997), we examine potential domains for interventions to address MPA using a dual ecological framework. Developed in roughly the same period, Gibson’s (1979) ecological psychology and Bronfenbrenner’s (1979) bioecological theory offer complementary perspectives on how individuals are continuously shaped by, and in turn shape, the environments in which performance takes place.

First, Gibson’s ecological psychology conceptualizes perception and action as inseparable, emphasizing that individuals move and respond within environments that offer affordances—perceived possibilities for action that depend on their abilities, goals, and skills (Gibson, 1977, 1979; Rietveld & Kiverstein, 2014; Stoffregen & Bardy, 2001). Applied to musical performance, this perspective highlights how performers attune to the affordances of the stage: acoustic conditions, audience behavior, interactions with co-performers, instrument characteristics, and performance conventions. MPA can thus be understood not only as an internal state, but as a disturbance in this process of attunement, where the performer’s perceptual and motor systems struggle to organize action fluently in relation to situational demands. In ecological terms, questions about MPA therefore become questions about how far performance environments support or disturb this ongoing attunement to affordances (Clarke, 2005; DeNora, 2017; Reybrouck, 2012; Waters, 2007; Davis, 2011).

Second, Bronfenbrenner’s bioecological theory situates individual development within nested systems of social interaction, ranging from the microsystem (e.g., family, peers, teachers) and mesosystem (relations between these settings) to the exosystem (institutional, organizational, and policy contexts) and macrosystem (cultural norms, values, and ideologies), all unfolding over time within the chronosystem (Bronfenbrenner, 1979; Bronfenbrenner & Morris, 2006). This model allows us to trace how musical self-concept and MPA are co-constructed across educational pathways (Davidson, 2002; Davidson et al., 1997; O’Neill, 2017), institutional cultures in conservatoires and professional music-making (Gaunt et al., 2021; Green, 2002; Schippers & Grant, 2016), and broader, often implicit performance ideals—such as the virtuoso performance model (Jorgensen, 2003; MacDonald & Saarikallio, 2024)—that shape shared expectations of what counts as successful performance. Insofar as this model privileges exceptional, flawless achievement and constant comparison, it can function as a holistic cultural–ecological frame, heightening the stakes of performance and increasing vulnerability to MPA. Within this perspective, preventive interventions do not target only individual symptoms, but also the design of learning environments, assessment practices, and institutional priorities (Kelly, 1967/1968, 2005; Donovan & Spence, 2000; Webster, 2003; Gaunt et al., 2021; Hess, 2019).

### 1.2. Aim and Research Questions

Grounded in this dual framework, we examined how these interwoven dynamics shape the occurrence and management of MPA within music performance ecosystems, with particular attention to identity processes in Western classical music contexts. Specifically, we addressed the following research questions:

1. What interactions in music performance ecosystems shape the experience of MPA?
2. How are these interactions related to the performer’s musical self-concept?
3. How can the interplay of social, educational, cultural, technological, and other environmental factors contribute to the formation of ecosystems that reduce the risk of developing MPA?

## 2. Materials and Methods

### 2.1. Participants

Eleven musicians participated in the study—five men and six women, aged between 23 and 62, with professional music careers ranging from one to 37 years. The participants included eleven performers (see Table 1): three pianists, two violists, two solo singers, a violinist, a cellist, a flutist, and a harpist.

Participants were selected on the basis of their career resumes, with the basic requirement being a distinctly stage-oriented musical career with experience in solo, chamber, and orchestral performances. The second selection criterion was their success in these roles, as reflected in their professional engagement in institutions of wider public importance, such as radio and philharmonic orchestras, national opera houses, public universities, and national music competition networks. In selecting participants, we took into account not only their formal achievements but also their activities in less formal forms of musical engagement.

### 2.2. Data Collection

The procedures used in this study complied with the European Code of Conduct for Research Integrity, the Declaration of Helsinki, and the Personal Data Protection Act. Ethical approval was granted by the University of Ljubljana Ethics Committee (UL KERL; No. 054-2025). Written informed consent was obtained, participation was voluntary with the option to withdraw at any time, and no incentives were offered. Sampling took place from April to May 2025. Candidates were identified from the research framework and professional biographies, prioritizing social presence and recent public exposure (e.g., major concerts; long-standing collaborations with leading institutions; flourishing/winding down of careers; notable transitions). Following ethical approval on 12 June 2025, the researchers personally contacted the invitees. Some contacts were derived from shared professional experience, while others were obtained via institutional mailing lists. Initial outreach (via telephone or email) provided detailed study information, followed by formal invitations. The plan was to include 12 participants, but scheduling for one invitee stalled due to workload, thus yielding a final sample of 11.

The interviews (13–29 June 2025) were audio-recorded and conducted in a conversational manner. Seven were conducted online (MS Teams, *n* = 5; Zoom, *n* = 2) due to concert/work commitments or because participants were residing abroad. In-person interviews, at the participants’ request, took place in their homes, workplaces, or neutral public venues. In-person interviews were, on average, three minutes shorter than those conducted online, and the overall duration ranged from 40 to 95 min. Recordings were transcribed verbatim in colloquial Slovenian.

### 2.3. Interview Protocol

Data were gathered via semi-structured interviews built around eight biographical-narrative questions focusing on social interactions and the meanings participants ascribed to musical development, identity formation, and MPA, with performing as the core experiential arena. The guide covered (1) earliest performance memories (family/early settings); (2) changes across music school–conservatory–academy; (3) effects of professionalism; (4) language used for pre/during-performance feelings; (5) positive processes/strategies/practices in school, cultural, and institutional environments; (6) influential situations shaping beliefs/attitudes/self-assessment; (7) changes across career transitions and their drivers; and (8) performance setups that reduce stress and support well-being across education levels and professional practice, including personal approaches. The full semi-structured interview guide is provided in the Supplementary Materials (File S1: In-Depth Interview Questions).

### 2.4. Data Preparation and Analysis

Transcripts were prepared and coded in Microsoft Word; Excel supported administrative organization (code lists, participant–theme matrices, summary tables). Two researchers coded the corpus in a complementary split (~40%/~60%) using open, line-by-line coding with memo writing (Charmaz, 2006). After an initial pass, a comparison meeting resolved overlaps and clarified code boundaries. Coding then proceeded by negotiated consensus with explicit inclusion/exclusion rules and examples. This yielded 67 codes, subsequently organized into a thematic network: codes were clustered into 11 themes, grouped within four macro-themes.

The analytic stance was abductive, iterating between data and the theoretical proposition. This approach reflects constructivist grounded theory principles—iterative comparison and memo-based theorizing (Charmaz, 2006)—and aligns with the negotiated-agreement tradition in Consensual Qualitative Research (CQR), emphasizing team consensus and an explicit audit trail rather than coefficient-based reliability (Hill et al., 1997; Hill, 2012).

### 2.5. Rigor and Validation

Triangulation comprised testing and reformulating theoretical starting points against empirical data and continuous coordination between researchers to monitor saturation and thematic-network coherence. A member check was conducted: transcripts were returned to the participants for verification, and the findings were jointly reviewed (Thomas, 2017). On this basis, we constructed a grounded theory (Charmaz, 2006) explaining how the participants experience factors related to MPA within performance as a broader sociocultural phenomenon. To protect privacy, the participants were assigned pseudonyms in all research materials and analyses; during transcription, identifiable details (e.g., names, precise locations) were omitted or simplified.

## 3. Results

The music performance ecosystems we examined revealed a network of contextual content for managing MPA, which we grouped into four primary themes in our results.

**Figure 1:**
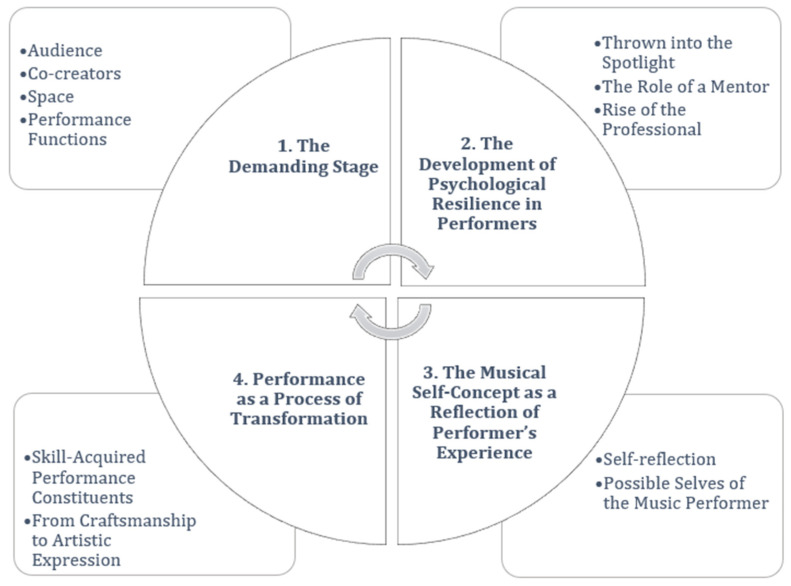
Music Performance Ecosystem: macro-themes and themes.

Each macro-theme represents a core set of content, within which a series of themes intertwine to provide a deeper understanding. These themes are interconnected processes that, together, form a sociocultural view of coping with MPA, as shown in Figure 1.

In Figure 1, four macro-themes and their corresponding themes are shown: (1) The Demanding Stage (themes: Audience; Space; Co-creators; Performance Functions); (2) The Development of Psychological Resilience in Performers (themes: Thrown into the Spotlight; The Role of a Mentor; Rise of the Professional); (3) The Musical Self-Concept as a Reflection of the Performer’s Experience (themes: Self-reflection; Possible Selves of the Music Performer); and (4) Performance as a Process of Transformation (themes: Skill-Acquired Performance Constituents; From Craftsmanship to Artistic Expression).

The model articulates four interlocking macro-themes that map the performer’s developmental and situational landscape. *The Demanding Stage* highlights how ecosystemic pressures converge onstage, while the *Development of Psychological Resilience in Performers* scharts the long arc through which family, education, practice, and mentoring transform early exposure into coping resources. *The musical self-concept as a reflection of the performer’s experience* frames identity work as an ongoing negotiation—“What am I like?” and “Who am I becoming?”—that is continually tested in performance. Finally, within *Performance as a Process of Transformation*, skill-acquired performance constituents—integrated aims and resources—are aligned through attunement, culminating in artistic expression that transcends craftsmanship.

### 3.1. The Demanding Stage

A musical performer who prepares for a performance with their instrument always steps onto the stage with an awareness of the unpredictable. Despite lengthy planning and rehearsals, they are faced with a complex and simultaneous intersection of impulses. These arise from the intertwining of factors determined by the audience, the space, the co-creators, and the performance functions. These elements form thematic clusters in which key patterns are identified by individual categories, as shown in Table 2.

The network of factors shown in Table 2 often goes beyond the scope of advanced planning and requires a high degree of presence, responsiveness, and artistic flexibility. A significant web of interactions within this network involves the audience.

#### 3.1.1. Audience

Prior to stepping on stage, the performer anticipates the audience by forming internal representations of the expected interaction, which significantly influence their performance preparation. Upon going on stage, these are confronted and complemented by their immediate, dynamic interaction with the audience.

An unresponsive audience—*“that dead silence, now we’re quiet, we applaud a bit, and that’s it,”* as described by participant P1—often provokes heightened tension and rigidity in the performer’s expression. Participants seek to overcome this sense of emptiness through diverse strategies, including offering themselves rather than simply showcasing their skills and knowledge, providing verbal guidance during the performance, or initiating direct interaction with the audience. Such techniques create a more authentic connection that gives meaning to the musical event as a whole.

The motivational trajectory in relation to the audience allows us to observe a full spectrum of responses—ranging from negatively to positively oriented motivation—both of which directly affect the quality of the artistic performance.

On the negative side of the motivational bivalence, listeners most often play the role of evaluators or even selectors. This atmosphere is typical of exam situations, auditions, premieres, or other key performances where the performer feels intense pressure from professional judgment. This situation simultaneously fuels the desire to deliver a performance of the highest quality, while also potentially inducing “tightness,” as the performer becomes paralyzed by the fear of failure. The background of such feelings was explained by participant P8 as follows:

*“Playing for a panel—for someone who’s been sitting there for four or five hours as part of their job—I know it’s hard for them to listen to every next person, no matter how they play. It’s just not easy to sit there. And I find it really hard to play for that kind of panel.”*

On the positive side of motivational bivalence, supportive interactions—from imagined audience members to encouraging mentors—foster openness and strengthen the performer–audience relationship. According to participant P7, such interactions can lead to a shared musical experience:

*“That interaction with the audience, meaning it’s not just me and the piece I have to play as perfectly as possible, but I have started to open up more and more to the audience itself and to sense their reactions—that sometimes we were almost breathing together or were left breathless at certain moments.”*

The presence of professional colleagues also has a special influence, which performers often interpret as *“Attention! Musician in the audience”*—a warning signal that technical perfection needs to be increased, which some manage to do while maintaining interpretative relaxation, while others describe a reduced flow of interpretation.

The audience and the venue are interconnected factors through which performers seek new possibilities: *“It would be good to open up such spaces [for performing] where the audience could also be a bit more interactive.”* Participant P5 believed that space plays a crucial role in shaping the experience for both the audience and the performer.

#### 3.1.2. Space

The data suggest that the sound space is the foundation on which the performer builds their communication with the audience. To this end, they carefully prepare not only the flow of the performance but also explore the acoustic properties of the space that will best convey the sound of their instrument: *“Open-air concerts are often just something you ‘get through’… the venue dedicated to it makes the difference,”* said participant P2, who performs with his voice. Any acoustic ambiguity, accidental sounds from the instrument, or obstacles to the transmission of sound in the space contribute to the “nervousness” of the performance, as described by participant P4:

*“You can’t let any distractions throw you off—like someone coughing, people coming in and out, someone taking pictures up close, flashing lights, a detuned piano, the stage creaking, the piano bench squeaking, or the pedal squeaking…”*

The data suggest that environments idealized as symbolic spaces of exemplary performance—such as national opera houses, large prestigious concert halls with substantial audiences, and venues emblematic of artistic breakthroughs—further exacerbate MPA.

To alleviate the symbolic burden of these contexts, the participants stated that they enjoy trying out new performance spaces, which, with their informality, remind them of everyday social gatherings. They enjoy being connected with the audience in spaces that are otherwise intended for relaxed encounters. For a more authentic contact with the audience, they want to identify with them more, even if it is *“on the street”*—as this strengthens their performance confidence and psychological adaptation to the challenges of public performance. The participants believe that waiting for formally filled halls is misguided and that alternative spaces, such as streets, clubs, and interdisciplinary interiors, should be utilized to increase the number of performances, as this contributes more meaningfully to practice time. This common view was summarized by participant P2, who stated: *“If you have more performances, you somehow develop better. If you don’t have them, I don’t know if it [music learning] even makes sense anymore. Even if you have good knowledge, but don’t pass it on or share it with others—you’re kind of in an empty space.”*

With this in mind, they seek environments that allow for a more formative and less summative nature of performing. Participant P1, who first encountered this approach to performance preparation during university, explained this as follows: *“You’re already practicing performing even with pieces that aren’t fully prepared—you can even play just two pages, if that’s what you’ve learned, just so you get some experience, so you feel a bit of pressure, no matter how well the piece is actually prepared.”*

Our analysis indicates that this approach is feasible both within the educational system and in less demanding, informal performance settings. In such spaces, the participants felt it was permissible to reveal the “backstage” elements of performance, to improvise in order to overcome various technical difficulties, and to take a less uniform approach to clothing. In general, clothing is an important “secondary” factor that is closely related to the performance space. The participants are convinced that clothing “makes” the individual who steps onto the stage, but it can also constrain them, making it difficult to play an instrument and thus creating additional pressure during the performance. They supported themselves with specific preparations: *“I even practice in the shoes—and sometimes the dress—I’ll wear on stage, to get used to it. It adds a bit of pressure beforehand, like a mini run-through of the whole program”* [P1].

For example, a costume that is too heavy or cumbersome will restrict the opera singer’s freedom of movement throughout the entire opera performance. Taken together, the narratives suggest that the performer should therefore be an important decision-maker when determining what they will wear, the lighting, seating arrangements for orchestra members, air circulation, temperature, and other physical characteristics of the space that affect their working conditions.

#### 3.1.3. Co-Creators

Extensive preparations for a performance involve a great deal of solo practice, which must be blended with the joint performance of the co-creators on stage. This focus gives rise to several conflicts that can arise among orchestra members, between the accompanist and soloist, between alternates in singing roles, and so on.

Our grounded analysis revealed that a competitive mindset—seeking to outperform other musicians rather than adopting a cooperative approach—creates psychological tension. Participant P4, reflecting on the performative environment in which he developed as a musician, stated: *“…everything was a competition—every internal performance, every masterclass…”*

Participant P9 sought to transcend this dynamic through sincere, open communication: *“Just to talk, you know—when a difficult passage comes up, you complain a bit, and then it gets easier when you see that even the best one can’t quite do it either...”*

The atmosphere during performance—the “energy,” as the participants often called it—is transmitted among co-creators. As participant P9 put it: *“… when I’m sitting at the second stand, I really feel the people who aren’t used to sitting there—I feel them. I always feel that fear. And sometimes it spreads collectively. If the leader is insecure, that feeling transfers to the whole section.”*

Even the relatively inconspicuous performer—the tuttist, whose artistic freedom and expressiveness are subordinated to the collective—experiences significant stress. Constant adaptation and subordination give rise to a sense of constraint, a feeling of having one’s *“hands tied,”* [P8] whereby the performer’s interpretation goes unheard and their individuality unacknowledged.

Chamber music offers opportunities for greater personal expression, while at the same time, the in-depth collaboration between performers creates a more trusting environment, thus providing greater security. The participants are convinced that playing more frequently in smaller ensembles would increase the performer’s sense of autonomy and strengthen their self-confidence. This perspective was echoed by participant 5: *“The very approach to the work is already different—perhaps more relaxed. You also choose your colleagues on a different level; I mean, what’s better than playing chamber music with friends, right? That in itself makes you want to give a good concert; there are some nerves, especially if it’s difficult—it’s interesting… but the feeling of motivation is different”* [P5]. Approaches to creating an emotionally beneficial atmosphere among co-creators also include socializing on tours, guest performances, joint exercise, mediation, and sincere informal conversations.

#### 3.1.4. Performance Functions

The basic framework of a performance is also determined by its function. The participants understand this in terms of who they are performing for and for what purpose, as well as the goals they must achieve through their performance. At the same time, they question their own competence and vocation in this context. In this section, we present examples of the participants’ reflections on experiencing a high degree of responsibility, which, as noted by participant P1, also results in increased pressure.

*“…then they had to record me in front of the whole orchestra because they were recording a CD. I have to admit–that was huge pressure. You don’t want to disappoint the director, I mean the conductor, or the people who are recording, because then they’d have to do the take ten more times because of you, not to mention the others waiting to see if you did it well or not…”*

As a counterpoint, the same participant also described low-stakes, background engagements where evaluative pressure is minimal and technical demands are modest:

*“When I know it’s just an event, some pop thing… I don’t worry much about the performance. I once played for two hours as guests were arriving—it’s the easiest kind of performance. It’s not that technical, and even if something goes wrong, it doesn’t matter; no one is listening closely”*

[P1].

The performer feels a sense of responsibility for their artistic mission, which encompasses expectations from the composer, mentor, collaborators, themselves, the employer, the client, and ultimately the audience. The conglomeration of standards that this creates in the performer’s mind determines the goals of the performance. The spectrum ranges from musical interpretative elements to the degree of their perfection, as well as to the empathy and message conveyed to both their collaborators on stage and the individual members of the audience. The participants described responsibility as a key emotion that creates the solemnity and seriousness of the moment, even before the performer sets foot on stage. Awareness of their creative mission, shaped through years of training, also imposes a demanding task on the performer—to express it satisfactorily. The greater the public recognition, the greater the responsibility and, consequently, the pressure on the performer. Some participants also believe that this increases with years of experience. Relief is possible by *“switching off”* from an overly intense and emotionally engaged experience of the performance. However, with this strategy, the participants often limit themselves to simple, more superficial requirements and tasks that involve clear and less complicated executions. The use of *“autopilot”* [P10] for example, allows them to come off stage less tired and exhausted from the repeated experience of proving themselves and undertaking excessive emotional engagement.

Thematic analysis revealed that the purpose and goal of the performance influence its form and, consequently, the performer’s attitude toward performing. A broader understanding includes both formal and informal performance contexts, which are shaped by the previously defined characteristics of performance spaces and audiences. At various points on the continuum between formal and informal performance atmospheres, we can perform educational, ceremonial, entertaining, cultural, commemorative, experimental, traditional, and other types of performances. According to our participants, stage experience can function either as the *“icing on the cake”* [P3] or as a *“performance in progress”* [P1] in which a program is presented before it has reached the desired level of performative refinement. The level of relaxation experienced in each performance situation is shaped by a combination of individual traits and the overall atmosphere accompanying the event. In some cases, performative purposes and goals construct a rigid and uniform environment, placing the performer under behavioral strain. As participant P2 reflected:

*“So, I was really nervous. And I had to play in the G. Hall, then they told me the president of the country would come, and that after the concert I’d have to put on a tie and go meet him, and all that stuff. After that performance, I wondered if I even wanted to do this.”*

### 3.2. The Development of Psychological Resilience in Performers

Based on the participants’ accounts, the developmental trajectory of a musical performer who cultivates psychological resilience through performance practice can be delineated into three main phases, organized under the following themes: thrown into the spotlight, the mentor’s role, and the rise of the professional. These phases are explained through the categories presented in Table 3.

#### 3.2.1. Thrown into the Spotlight

Performing is associated with a family environment that is traditionally oriented toward singing and learning instruments. It was there that the participants first experienced the expectations of a random audience eager to hear them perform. This made them feel proud, but also embarrassed, as what should they perform? This feeling stayed with them even when they became professionals, when they still found it hard to pick the right piece from their vast repertoire for a “promotional” performance. It had to be well prepared, polished, and suitable for the audience. This was especially difficult for the participants during their student years, when—based on their mentor’s decision—they focused only on etudes and technical issues, such as bowing. When they were invited to play something, they did not have an appropriate repertoire prepared, which deepened their sense of limitations in interpretation. In primary education, the taken-for-granted performativity and the associated didactic emptiness were described by participant P9 as follows:

*“I was simply faced with the fact that I had to perform, so I got up there. And however it goes, it goes.”* Participant P3 statement points to an institutional gap: *“In the education system there isn’t much emphasis on how to cope with stage fright—we’re left to our own devices.”*

Participants described somehow getting through these experiences and overcoming their MPA, hoping that the audience did not notice their nervousness. After coming off stage, they were glad that their suffering had at least earned them social recognition, if not praise from their mentor.

However, the adolescent crisis deepened the affective phenomena experienced during performances, leading the young musician to make new decisions. Participant 9 describes their quiet struggles with MPA as follows: *“Just before the events themselves, there was always stress in the form of diarrhea and vomiting. It was still happening at the academy. But I always managed just fine. No one knew I had stage fright… I remember looking for a way on my own to get rid of that negative stage fright”* [P9]. At the same time, educational settings often promote irregular, high-stakes exposure, which undermines gradual desensitization: *“We practice and practice…one performance a year… then straight to an audition. That’s not natural,”* said participant P8. In support of this view, participant P1 emphasized the value of gradual desensitization through frequent low-stakes classroom recitals:

*“Classroom recitals would really help: the more often you run a piece—also at different stages of mastery—the more confident you become, the better you know how to react, and the clearer the whole situation feels”*

[P1].

The participants described critical experiences from performances that provoked disappointment, discomfort, “brooding” or rumination, increased reflection, and increased intrapsychic and environmental demands. As part of the latter, in recent years, a growing awareness of the social role of the musical performer has developed, with the mentor playing an important role in this context, as discussed below.

#### 3.2.2. The Role of a Mentor

The role of a mentor can also be partly played by parents, conductors, fellow students, or professional colleagues, but it is best fulfilled by an instrument or singing teacher. Where performance education was limited in childhood, adolescent mentoring often placed heightened emphasis on performance, focusing on mastering the musical craft.

Because this period in a young musician’s life involves numerous exams and other performances where they must meet certain standards, the mentor tends to be primarily concerned with fostering technical excellence. As participant P5 recalled: *“I had already done an audition for a solo with orchestra at the Academy… And in a way, of course with some guidance from my professor, I was dealing with major problems in the third movement, because my wrist was blocked and again it wouldn’t go at the right tempo. And I practiced the whole summer in a slow-motion manner, every movement, so that it started to move a little.”* Even so, the lack of explicit support during training did not deter participants from pursuing music careers, as they continued into advanced study and professional work. The unresolved tension that accumulated in the process was resolved independently, or with the help of more advanced university programs during their studies and later in their professional lives.

As participant P9 stated, while also expressing her hope that things had now improved: *“I found a book [during studies at university] that talked about autogenic training. I started doing the exercises, and I have to say, it was a real discovery for me at the time. A discovery—because no one, not even the professors, had introduced us to anything like that, on how to deal with MPA. We didn’t receive any knowledge about it at school. I really regret that. I hope it’s better now. I do hope they have some kind of course on preparation.”*

Beyond technical instruction, mentors serve as an emotional guide for performers—either by providing a secure base *(“The main-subject teacher was a wonderful person—warm, safe, and encouraging,”* [P6]) or, conversely, by leaving an affective vacuum that students must navigate alone *(“… At the Academy I missed emotional support … I started to shut down instead of express myself”* [P3]). Although most mentors did not directly and systematically address the taboo of MPA, they left a strong emotional mark on the participants, shaped by their love of music. The mentor’s brief instructions before performances—such as *“On stage, you have to feel like the best singer in the world,”* or *“You know it, you can do it-good luck!”*—offered the mentees a direction along which they instinctively developed as performers. The mentor’s praise meant a great deal to them and had a positive effect on their stage confidence. The circle of satisfaction was complete when the diligence of the learning performer brought satisfaction to the mentor and a successful performance. However, it became increasingly clear that MPA would not disappear, but would have to be tackled more systematically and comprehensively.

#### 3.2.3. Rise of a Professional

Social breakthrough consists of a series of key appearances on stage through which participants overcome various obstacles on the path to victory, leading to the decision that performing is something they want to pursue professionally.

Participant 11 experienced this as a holistic social task: *“I auditioned for the opera chorus. That was the biggest turning point for me… I was now employed, and at the time I was really afraid: how would I manage in L. [big city]? I come from the periphery. How would I deal with all those people who were already, you know, established in that milieu—people from a different background?”* [P11]. Motivated by responsibility, recognition, and success, the participants gradually took on roles that brought them musical leadership and exposure, even though some believe that they are actually introverted people who do not like to be in the spotlight. *“At first I was employed as a tutti player. After five years I auditioned again and moved to a deputy principal position—quite unexpectedly”* [P9]. The episodes that marked the rise of a publicly recognized performer followed one another, from schooling and professional trials to less formal, often genre-diverse confirmations, which, as a rule, brought a positive change to their careers.

Becoming a professional performer meant a final confrontation with a representative environment in which it is necessary to update and perfect their stage experience on a daily basis—a fight to the end. They have thus accepted a lifelong commitment to practice, while seeing the discomfort they experience before a performance to the price of a successful concert. Participants describe their discomfort as negative stage fright, nervousness, and stress, but they believe that this does not constitute a permanent anxiety disorder that would impair their quality of life. On the other hand, they devote a great deal of attention to their performances, arranging their rest and meal schedules, and even their family life is subordinated to the demands of performing. They often describe performing as very demanding, bordering on the impossible. The complexity of the musical text and high expectations for a perfect performance place an additional burden on them due to their lack of technique, the possibility of memory lapses, waiting to perform, and the high level of solo exposure.

**Figure 2:**
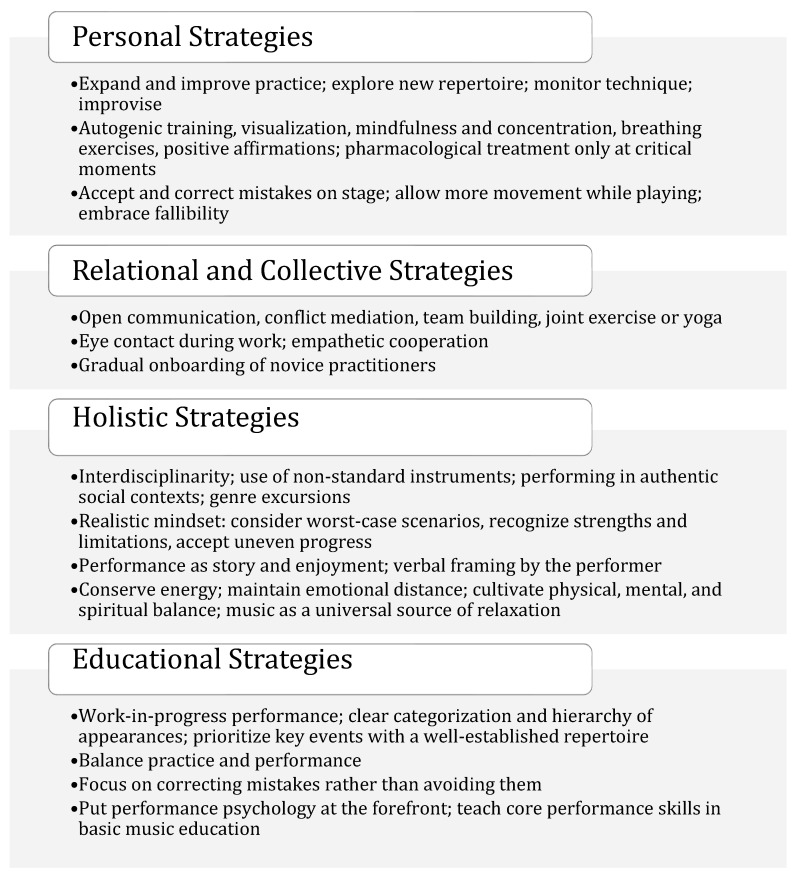
Experience-Based Coping Strategies for Managing MPA.

Figure 2 presents the personal, relational, collective, holistic, and educational strategies that the participants identified as leading to reductions in MPA, based on their experience or as visions for improvement in their work.

Performing, as well as introducing strategies for improving performance, testing them, and perfecting them, requires the participants to take a mindful and critical look at themselves. Based on self-reflective processes in musical self-concept, a socially recognizable expression of musical identity is developed.

### 3.3. The Musical Self-Concept as a Reflection of the Performer’s Experience

Self-awareness and psychological processing of performance experiences significantly guide musical performers both during performances and when planning their career paths. The categories listed in Table 4 highlight the themes of self-reflection and the possible musical selves of performers.

#### 3.3.1. Self-Reflection

*“It’s important that I take a moment to relax and tell myself that I’m ready. I listen to my body, give myself positive thoughts, and say to myself that it’s going to be okay.”*

[P10]

Participant 10’s account highlights the dual role of inner dialog as both a coping mechanism for pre-performance anxiety and a critical, self-evaluative voice during performance, positioning self-reflection as central to the performative process.

In the participants’ accounts, self-reflection served as a means to steer the course of performance, as they monitored their inner attitudes and self-judgments, and made changes in real-time. Through self-awareness of the accompanying affective processes—such as trembling, yawning, emotional excitement, confusion, tension, or breathlessness—the performers gain insight into existing MPA symptoms. They are then able to register these states and rapidly adjust their actions, often through an inner monolog.

Participant P3 recalled that at age 12 or 13, she suddenly realized:

*“…wait, what is this? Why am I suddenly shaking like this? And what if that happens to me right in the middle of the stage? What if things just start falling apart…”*

The benevolent inner “companion” of participant P1 knows the strategies to deal with such issues:

*“I won’t let this bring me down. Just focus, it will be fine. Just stay calm, just stay calm.”*

On the other hand, the critical inner voice functions as a specialized mechanism for evaluating performative moments. Participant P7 described his struggle to keep this voice in check as follows:

*“And of course, during the performance, that critical thinking kicks in: what are you going to do so it work… Yeah okay, that last bit wasn’t exactly brilliant… That inner critic starts chiming in with all sorts of ideas. It’s pretty uuuuuuuu [foreboding groan].”*

As performers mature, they accumulate countless impressions of both performance and preparation. This personal archive of experience gives the inner critic a more structured and authoritative voice—one capable of assessing success, pointing out mistakes, and also suggesting improvements.

However, since this inner voice primarily serves as the guardian of the holistic self, it also engages in (self-)criticism of its own strictness. We observed how the inner critic, originally “raised” as a perfectionist, gradually transformed into a more spiritual and benevolent commentator on musical performance, shaped by past experiences. As participant P1 stated:

*“…it went wrong, and I know how I could change it next time, but now I’m just going to keep playing…”*

Constant interpretation, explanation, and expression of views on the content of the performance are not only present on stage, but also characterize the preceding periods of preparation and planning, which become a way of life for professionals.

#### 3.3.2. Possible Selves of the Music Performer

*“So, a circus performer must not have breadth—must not allow it—because when doing that triple somersault, God forbid he thinks of anything else, or he’ll die. And for me, it’s the same during the Bartók concerto on the viola.”*

[P8]

Based on self-identification, the participants recognized various roles that accompanied and marked the development of a music performer. We list their names and definitions below:

1. The musician as a virtuoso, “circus performer”—narrowly defined roles, “shallow” dedication to producing flawless artistic performances.
2. The musician as a craftsman—conscientious and flawless, but an unemotional performer of a “commission.”
3. The musician as intellectual, artist—a comprehensively educated and spiritually awakened creator.
4. The musician as researcher—a role focused on the search for new challenges and ways of making music.
5. The musician as an athlete—a professional who complements their performing discipline and fitness with approaches from sport psychology.

Based on their experiences in these roles, the participants developed their own personal performance styles, which have had a significant impact on their musical identities. A fairly similar educational background, combined with further maturation, individual experiences, and self-reflection, led to different jobs and interests among the participants. Although much of the data pointed to the formation of an artistic-interpretive, even solo career path, the participants’ biographies often revealed a departure from prominent positions and a shift toward research, social activism, the search for new educational paths, and spiritual and intellectual enrichment.

However, the conglomerate of roles that help to create a convincing performer on stage requires the constant maintenance of distance, or reciprocity, between the stage persona and the holistic self. One participant, who has performed more than 60 different roles—including *Nabucco* over 100 times—repeatedly emphasized the importance of realistic self-awareness: *“I have always proceeded from the premise that I was never, ever Nabucco or Rigoletto or any other character, even when I stepped off the stage…. Because that’s the problem. The problem is that then you’re still Rigoletto, and there will always be one person or one character who is above you. I always stepped off the stage as MK. I am a real person.”*

In addition to the importance of self-reflection, this quote also points to the ability to transform that a musical performance in different contexts elicits and demands from the performer, which is the starting point for our last macro-theme.

### 3.4. Performance as a Process of Transformation

The fourth macro-theme highlights the radical transformation of concepts in the dramaturgical arc of performance. The content of the transformation is centered on the categories and themes presented in Table 5.

Within Performance as a Process of Transformation, Skill-Acquired Performance Constituents—integrated aims and resources—underpin the musician’s shaping of a performance concept; through attunement, this can resolve as artistic expression, marking the shift from craftsmanship. The analysis that follows traces these dynamics in chronological order.

#### 3.4.1. Skill-Acquired Performance Constituents

The participants experience the moments before a performance as anticipation, confrontation, taking control of events, execution, and sharing the universal value of music with their co-creators and the audience. Some enter a “bubble,” others seek “homeostasis,” balancing “energies” during performance—all grounded in Skill-Acquired Performance Constituents, that is, resources and aims shaped by many hours of preparation.

We identified the participants’ internal resources for preparation as physical, emotional, cognitive, academic, and spiritual. Physically, participants relied on day-of routines and rest: *“On the day of a performance I’d sleep a bit more, sing through the whole role before lunch, then rest; an hour before the show I’d go to the dressing room—no more singing—just lay out the score, review the libretto, calm down. That was my routine. Always”* [P11]. As P9 emphasized, pairing deliberate rest with brief, goal-directed cognitive checks were pivotal for sustaining focus: *“I never practiced physically on the day of a concert … visualization … because I wanted to be rested. It worked brilliantly for me”* [P9]. Emotionally, performers kept a prospective map of feelings shaped by audience, setting, and repertoire; in opera, grounding in the libretto primed expression—*“Librettos teem with emotionally charged words and actions … you respond differently—through the voice and, above all, through facial expression”* [P11]; in chamber contexts, *“…chamber playing saved me—I really enjoyed it”* [P6]. Academically, they safeguarded fluency through focused technical and score work: *“…and practice is not just sitting at the instrument; you can practice all day simply by analyzing the score and preparing the notation”* [P1]. Spiritually, they re-anchored the holistic idea of the performance before going on stage: *“I still remember standing there, looking out the window to focus. I must have looked like a little statue”*, a moment participant P5 described as the need to *“pull himself together at a higher level”* before stepping on stage. The interaction of these resources was described as supporting the achievement of planned performance goals, the most frequently mentioned being technical accuracy, virtuosic display, aesthetic experience, empathetic co-creation, and the sharing of music. Some participants believe that the desire for a flawless academic performance distances them from relaxation and the deeper meaning of performing, and thus they are increasingly focusing on performing music as an artistic product that they give to their co-creators and the audience. *“The key shift is toward mission—what I want to contribute with my music, what I want to give to others—away from the notion that I have to be the best, perfect, in the spotlight”* [P2].

#### 3.4.2. From Craftsmanship to Artistic Expression

The participants have been preparing for a “special experience” throughout their lives—in school, during rehearsals, and just before stepping on stage. These actions were intended to open the invisible channels and mediums that transform technical craft into artistic structure, an experience that the participants described as a calling to create, most clearly sensed on stage. However, instead of the intended academically persuasive performance, the rendition sometimes devolves into bodily and cognitive symptoms—*“…your throat tightens… You have a dry mouth; you’ll forget the text simply because you don’t know whether that high note will go through or not”* [P11]. The performance is then no longer under the performer’s control: *“my concentration went completely on its own … I was just waiting for it to be over as soon as possible”* [P1]. In extreme cases, an actual “collapse” of the performance occurs—*“I stopped … I had to stop”* [P5, memory block at a competition].

**Figure 3:**
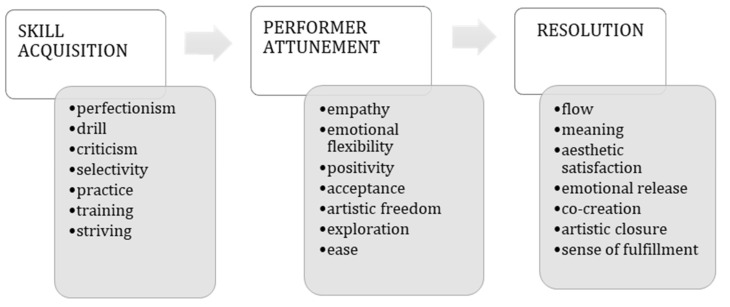
The Shift Between Skill Acquisition and Performer Attunement in the Transformation of Craft into Artistic Practice.

Figure 3 illustrates how this shift between the development of skill acquisition to performer attunement is experienced. Attunement—described here in terminology grounded in our data—can be seen as a key transformational phase, an alignment between the performer’s internal resources and the expressive demands of the performance, which may or may not culminate in resolution.

Participants who found themselves in the very core of performance-related conflict describe it as a web of global incongruities between the preparatory processes leading up to the performance, and the spontaneous expression triggered by the performer’s instinctive input, often driven by an opposing conceptual orientation. Participant P6’s thoughtful reflection offered a deeper look into this:

*“Perfectionism is incredibly present. You have to become aware of what really matters. Is it important to play all the notes precisely and in perfect rhythm—or is there more to it? Is it important how warmly you play, what kind of ‘air’ your phrase carries? … Does the phrase have direction, a line, or is everything static and without feeling—like you’re just pushing keys on a machine? Do you even like what you’re playing? Are we enjoying it? Does it have character? … I wanted to shift the focus to this—not just to notes and a metronome. Of course, that’s the foundation, but that’s practice, drill… That’s another kind of development. That’s not an analytical mind, it’s different layers—the body, the feeling, the senses… It’s about an aesthetic sense that has to live inside you.”*

The disjunction between the processes that shaped the performer’s perfectionist identity is already hinted at during the preparation phases, but it becomes fully apparent during the performance itself. At this point, it appeals to the empathetic interpreter to “forget” the logic of drill-based training and shift focus to emotional expression.

The act of performing invites the musician to transform critical self-monitoring into pure acceptance and to let go of the practice-based principles that had underpinned their technical competence. These competencies must be transmuted into artistic boldness and expressive freedom. Similarly, the “disciplining” nature of musical training—sometimes likened to conditioning—must, on stage, be counterbalanced by an exploratory equivalent, ideally embodied as playful ease rather than a lifelong burden of effort.

The dramaturgical resolution of such performative dynamics very occasionally culminates in a flow experience, which participant P3 interpreted as a successful stage transformation:

*“I once had a performance at the Academy of Music where I practically stepped out of my own body. I wasn’t here anymore—I saw myself from the outside… Everyone was amazed because it was an exceptional performance. I remember sitting at the piano, but I was up here somewhere, you see, and I was still functioning, but at the same time, I wasn’t really there, right?”*

It becomes evident from participant P11’s statement that achieving a state of flow is contingent on co-creation.: *“You don’t need a single word—it’s the glances, the music, this kind of symbiosis on stage… I don’t even know, it’s like a whole conglomerate, so many things have to align for it to really happen.”*

Participant P3, on the other hand, highlighted the importance of technical and emotional readiness as a prerequisite for flow: *“In my body, I felt absolutely confident at that moment—in the flow of the performance. Yes. I could completely let go because I fully trusted my body, my emotions, and then it just happened.”*

It becomes clear that flow is only attainable when the performer has engaged in long-term internal preparation: extensive practice, rest, mental clearing, and the development of personal strategies. These resources are then actualized during performance, not as a collection of mastered skills, but as a pure artistic language. However, elevated artistic expectations can sometimes result not in transcendence, but in collapse—a total loss of control followed by deep disappointment when the performer fails to realize any part of their envisioned outcome.

The younger participants in this study are increasingly being educated within a paradigm that values not only technical proficiency, but also the flexibility needed to transform units of knowledge into interpretive elements. During their studies, they come to recognize that practice entails more than mechanical repetition, that shortcuts can be valid, and that they must become autonomous architects of their own performance practice. To do so, they must also accept performances that may not resemble monuments of musical literature, but instead align with the process-oriented underpinnings of their performative identity. Or as participant P6 put it:

*“This is a long-term, integrative development. You have to connect many things just to get close to great music.”*

## 4. Discussion

### 4.1. Summary and Implications of Results

This study investigated how relational dynamics within Western classical music performance ecosystems shape MPA and performers’ self-concept, and how environmental interactions within these ecosystems may help reduce its risk.

From an ecological standpoint, our study reveals four interlocking macro-themes that circulate rather than unfold linearly, as onstage experience continually reshapes self-concept and preparation practices, which in turn feed back into the stage.

In Gibsonian terms, each venue offers a specific ecology of affordances—acoustics, sightlines, audience proximity, protocols, and symbolic prestige—that are relatively stable within a given event. From this perspective, what changes is chiefly the performer’s attunement to these affordances: how they perceive and use the possibilities for action that the situation offers, given their skills and goals (p. 8). Our data indicate that MPA emerges where there is a marked mismatch between the demands of these affordances and the performer’s available resources, for example in one-off, high-stakes auditions (p. 8) or in asymmetric expectations of tutti roles where expressive demands overshadow the performer’s own creative agency (p. 9). Cognitive-behavioral therapy (CBT) addresses this mismatch on the individual side by cognitively reframing rigid, inflexible concepts and by graded exposure (de Paiva e Pona, 2016). Our interviews also point to the ecological potential for bridging the gap: the factors that widen it are systemically addressable and were linked by participants to greater stability of attunement, such as chamber settings (p. 10, Cf. Section 3.1.3) with more autonomy and supportive ensemble climates (Ray & Hendricks, 2018/2019).

An ecosystemic approach recommends adapting affordances by introducing low-stakes audition steps (Spahn et al., 2016; Kegelaers et al., 2023) and by giving more attention to performance function and audience expectations in education and institutional performance contexts (Jorgensen, 2003). Our data highlighted underaddressed elements in preparation: What is the broader context of the musical presentation (p. 18)? In what function does the performer appear (Cf. Section 3.1.4)? Whom will they address and how (Cf. Section 3.1.1 and Section 3.1.2)? What is the significance of a particular segment of the performer’s skills and knowledge (p. 16, Cf. Section 3.3.2)? How and with whom will they prepare psychologically for the performance (p. 12)? Such structured preparation would not monopolize stage presence with virtuoso-technical persuasiveness alone, but would direct the performance toward the audience, purpose, and program curation, which are supported in the literature (Bennett & Ginsborg, 2018; Ginsborg & Chaffin, 2011; MacDonald & Saarikallio, 2024; Waddell & Williamon, 2022), and which the participants associated with reduced tension and more reliable attunement to the event. With this concept, the musical self-concept is addressed more comprehensively and in its manifestations—not only academically but also in bodily, cognitive, emotional, social, and, ultimately, spiritual fulfillment within each musical performance (MacDonald et al., 2017).

Viewed through Bronfenbrenner’s lens within the development of psychological resilience, communicative frames are shaped by gaps in microsystem interactions that should safely bridge the rehearsal–stage divide. The participants’ developmental narratives show that early exposure to performance was frequently inculcated without relational scaffolds (p. 12, Cf. Section 3.2.1), leaving performative skills insufficiently and inconsistently addressed in educational processes (Kenny, 2011). As a consequence, the participants reported that their most substantive conversations about fear in early music learning occurred in solitude; the inner “critic” assumed a regulatory role, leading to a profoundly solitary and disorienting phase of coping with MPA. Intervention efforts in educational settings should therefore prioritise regular early performance opportunities within nurturing, encouraging environments, which can foster a more joyful and expressive orientation to performing and help to minimise the later development of MPA (Osborne & Kirsner, 2022). In Bronfenbrenner’s terms, this implies that the early mentor–who occupies a central place in the performer’s microsystem through face-to-face tuition and mediates key mesosystem linkages between pupil, family, and institutional settings–needs to move beyond a predominantly technical focus to provide explicit performance support that can help build secure bridges and competencies for performance (Hays, 2013).

Based on our participants’ narratives, the transition to professionalism did not linearly eliminate anxiety; instead, it reconfigured it as strategies and meanings were rebuilt across transitions from study to work (see Figure 2)—a pattern also documented by Pecen et al. (2018). Within musical self-concept, performers continuously rework the demanding role of the musician—inflected by both intellectualism and “circus-manship” (see P8, quote: p. 15).

By the time our participants reach university and professional stages, they no longer ask whether they are “performers or perhaps teachers”; they experience the performer role as self-sufficient and unambiguous, in contrast to Pellegrino’s findings that teachers frequently oscillate between performer and educator identities (Pellegrino, 2009). Within this process of identification, a fine differentiation of possible selves unfolds, setting the terms of recognition for what counts as good performance (Papageorgi et al., 2007; Castiglione et al., 2018) and reframing MPA either as an identity tension (Schnare et al., 2012) or as a failed attempt at attunement (Clarke, 2005). Finally, our concluding theme is mechanism-focused: the shift from craft to art (Figure 3) integrates skill-acquired performance constituents into performer attunement and, where possible, resolution. This connects craft with art and aligns with live affordances (Gibson, 1979), understood as proximal processes within nested contexts (Bronfenbrenner, 1979; Bronfenbrenner & Morris, 2006), ecological accounts of musical meaning (Clarke, 2005), and the landscape of affordances/skilled intentionality (Rietveld & Kiverstein, 2014). When alignment succeeds (p. 18), performance tends toward a state of flow (Csikszentmihalyi et al., 2014); when it fails, drill logic dominates, characterized by low error tolerance and an affective “tightness” (p. 17). As detailed in “Performance as a Process of Transformation,” this mechanism brings out a vocational dimension: beyond the possible selves we identified a spiritual/expressive role appears in which the performer feels a universal mission to convey music—a move from academic proof to shared meaning (See P6 quote. p. 18 and P2 quote p. 17). This is accompanied by a mature detachment, allowing the performer, in real life, to step out of their stage shoes (p. 16).

Against this backdrop, our data delineate the first research question—what interactions shape MPA—across immediate on-stage couplings, early mesosystem conditions where exposure is normalized in the absence of supports, the quality of mentoring relationships and an ethos of “fight to the end”, which, from a longitudinal perspective, signal the performer’s social breakthrough over time (Creech & Hallam, 2011; Ray & Hendricks, 2018/2019). Regarding the second research question, these interactions train the inner voice and terms of recognition: competitive climates personalize anxiety and limited identity repertoire, whereas mentorship, collaborative microsystems, and more frequent, diverse—including informal—performance practices expand identity toward authentic expression (Juncos & Markman, 2016; Papageorgi et al., 2007; Pecen et al., 2018). Considering the third research question, key environmental levers that reduce MPA risk include graded exposure/desensitization in education (Kenny, 2005), institutionalizing multidimensional strategies already in use (Figure 2), and less rigid pedagogical preparation, which includes informal and non-formal performance opportunities and music-activism initiatives (Hess, 2019) oriented toward the ecology of attunement.

With a sustained ecological approach to MPA, we can clarify what lies behind the mentor’s *“You know it, you can do it—good luck!”*: the conditions that allow craft on stage to become artistic expression—so the performer will not be merely a “lucky one,” but clearly the result of successful attunement.

#### 4.1.1. Theoretical Implications

Overall, our findings indicate that MPA is best understood not as an individual pathology, but as an ecological response shaped by interactions with the environment. In line with Gibson’s (1977) concept of attunement, performers’ perceptual and emotional responsiveness to performance affordances plays a central role, while Bronfenbrenner’s bioecological model (Bronfenbrenner & Morris, 2006) highlights how systemic layers—from immediate social interactions to broader cultural contexts—co-create developmental trajectories and resilience.

#### 4.1.2. Practical Implications

From an ecosystems perspective, attunement can be cultivated by opening rehearsals to audiences and introducing early “good-enough” performances; adopting pedagogies that weave together formative and summative elements, foster flexibility, and normalize imperfection (Juncos & Markman, 2016). Moreover, improvisation should be used deliberately as training for real-time adaptation and pressure management (Biasutti & Frezza, 2009; Larsson & Georgii-Hemming, 2019). Across the educational pathway, the repertoire should be aligned with developmental stages and exposure paced so that the degree of challenge increases without overwhelming a performer’s capacity. Preventive strategies include:

- introducing psychological performance skills early in training,
- balancing practice and performance opportunities,
- scaffolding with repertoire suited to developmental stages, and
- gradually increasing exposure to high-stakes situations (Kenny, 2005).

Such measures, supported by institutional policies (e.g., tutoring systems, protective environments), could reduce the risk of developing MPA.

### 4.2. Strengths and Limitations

A strength of this study is its innovative ecological framing of MPA, integrating social, cultural, and psychological factors. Biographical-narrative interviews yielded rich qualitative data, supported by grounded theory methods that ensured empirical grounding. The sample’s diversity of age, gender, instrument, and career stage also enhances cross-contextual insights.

Limitations include the small, single-country sample (N = 11), which constrains transferability beyond Western classical traditions. The use of retrospective self-reports raises potential recall bias, and both authors’ positionality in performance/pedagogy may have influenced interpretation. The analysis was conducted by a two-researcher team using CQR and without an independent audit, although we partially mitigated the risk of bias through member checking. Finally, the absence of longitudinal data limits understanding of how MPA and resilience evolve across careers.

### 4.3. Future Directions

Future work should directly investigate onstage transformation, with a focus on cognitive, emotional, and behavioral processes. Longitudinal and mixed-method designs would help track the evolving interplay among self-concept, performance contexts, and well-being. Programs such as FRIENDS (Ahlén et al., 2012; Webster, 2003) warrant investigation in music education as potential ecologically oriented and resilience-building interventions for preventing MPA. In particular, core FRIENDS strategies—such as guided discussions about anxiety in performance situations, cognitive restructuring of catastrophic performance thoughts, practicing breathing, relaxation and structured peer-support activities—could be adapted and integrated into both individual lessons and group classes as a regular part of music learning and performance preparation (see Gill et al., 2024 for examples).

## 5. Conclusions

This study views MPA as a dynamic, ecological process shaped by multiple social and developmental factors. Performance unfolds within complex interactions among the musicians, mentors, peers, and cultural contexts. MPA arises not just from technical challenges but from navigating one’s identity, relationships, and the environmental demands on stage.

Across different educational and professional stages, performers face unique pressures. Early training often lacks consistent social support, leaving young musicians to manage MPA largely on their own. Mentors mostly tend to focus on technique, while emotional and identity development around performance remains under-addressed.

A key insight is that effective performance depends less on rigid control and more on attunement, in particular, being flexible and responsive to the moment and environment. This shift from perfectionism to greater coherence between formative and summative educational strategies in performance supports authentic artistic expression and reduces MPA. Integrating improvisation and behavioral–cognitive techniques for performance preparation into music education can enhance this process. Practically, institutions should foster protective environments by moderating risk factors, balancing practice with psychological skills training, and promoting gradual exposure to performance challenges to build resilience and support healthy artistic careers.

Traditionally, the virtuoso performance model has focused primarily on the importance of knowing—“you know it”—and practicing—“you can do it.” However, in managing MPA, we should also acknowledge the need to transform certain cognitive and behavioral patterns developed through the learning process. To overcome this gap, sometimes manifested as a brief wish before performing, such as “good luck,” we should begin taking empirical steps to define and measure certain entities, which for now remain at the level of an instinctively shaped performing mindset.

## Figures and Tables

**Table 1:** Demographics of the Participating Music Performers.

Code (Performer/Number)	Gender, Age	Instrument	Years of Performing Career
P1	F, 23	Harp	1
P2	M, 28	Voice	10
P3	F, 42	Piano	22
P4	M, 42	Piano	22
P5	M, 44	Violin	21
P6	F, 49	Flute	29
P7	F, 49	Piano	26
P8	M, 54	Viola	32
P9	F, 55	Viola	31
P10	F, 58	Cello	34
P11	M, 62	Voice	37

F = female, M = male.

**Table 2:** The Demanding Stage: Themes and Categories.

Themes	Categories
Audience	Interaction Motivational Bivalence
Space	Sound Space Idealized and Symbolically Significant Environments Creation of New Performance Spaces Working Conditions
Co-creators	Cooperation/Competition Chamber Music—Personal Expression
Performance Functions	Responsibility for the Artistic Mission Purpose and Goal of Performance

**Table 3:** The development of psychological resilience in performers, themes, and categories.

Theme	Categories
Thrown into the Spotlight	Family Tradition Taken-for-Granted Performativity
The Role of a Mentor	Musical Craft Mastery Emotional Guide
Rise of the Professional	Social Breakthrough *“Fight to the End”*

**Table 4:** A performer’s musical self-concept as a reflection of performer’s experience, themes, and categories.

Themes	Categories
Self-Reflection	MPA Symptoms Inner Critic
Possible Selves of the Music Performer	Roles Holistic/Stage Self

**Table 5:** Performance as a process of transformation, themes, and categories.

Themes	Categories
Skill-Acquired Performance Constituents	Resources Goals
From Craftsmanship to Artistic Expression	Attunement Resolution

## Data Availability

The original contributions presented in this study are included in the article/supplementary material. Further inquiries can be directed to the corresponding author.

## Supplementary Materials

The following supporting information can be downloaded at: https://www.mdpi.com/article/10.3390/bs15121696/s1, File S1: Semi-Structured Interview Guide (In-Depth Interview Questions).
